# Accuracy and Resolution of Kinect Depth Data for Indoor Mapping Applications

**DOI:** 10.3390/s120201437

**Published:** 2012-02-01

**Authors:** Kourosh Khoshelham, Sander Oude Elberink

**Affiliations:** Faculty of Geo-Information Science and Earth Observation, University of Twente, P.O. Box 217, Enschede 7514 AE, The Netherlands; E-Mail: oudeelberink@itc.nl

**Keywords:** range camera, calibration, sensor, RGB-D, point cloud, triangulation, imaging, error budget, laser scanning

## Abstract

Consumer-grade range cameras such as the Kinect sensor have the potential to be used in mapping applications where accuracy requirements are less strict. To realize this potential insight into the geometric quality of the data acquired by the sensor is essential. In this paper we discuss the calibration of the Kinect sensor, and provide an analysis of the accuracy and resolution of its depth data. Based on a mathematical model of depth measurement from disparity a theoretical error analysis is presented, which provides an insight into the factors influencing the accuracy of the data. Experimental results show that the random error of depth measurement increases with increasing distance to the sensor, and ranges from a few millimeters up to about 4 cm at the maximum range of the sensor. The quality of the data is also found to be influenced by the low resolution of the depth measurements.

## 1. Introduction

Low-cost range sensors are an attractive alternative to expensive laser scanners in application areas such as indoor mapping, surveillance, robotics and forensics. A recent development in consumer-grade range sensing technology is Microsoft’s Kinect sensor [1]. Kinect was primarily designed for natural interaction in a computer game environment [2]. However, the characteristics of the data captured by Kinect have attracted the attention of researchers from other fields [3–11] including mapping and 3D modeling [12–15]. A demonstration of the potential of Kinect for 3D modeling of indoor environments can be seen in the work of Henry *et al*. [16].

The Kinect sensor captures depth and color images simultaneously at a frame rate of up to 30 fps. The integration of depth and color data results in a colored point cloud that contains about 300,000 points in every frame. By registering the consecutive depth images one can obtain an increased point density, but also create a complete point cloud of an indoor environment possibly in real time. To realize the full potential of the sensor for mapping applications an analysis of the systematic and random errors of the data is necessary. The correction of systematic errors is a prerequisite for the alignment of the depth and color data, and relies on the identification of the mathematical model of depth measurement and the calibration parameters involved. The characterization of random errors is important and useful in further processing of the depth data, for example in weighting the point pairs or planes in the registration algorithm [17,18].

Since Kinect is a recent development—it was released in November 2010—little information about the geometric quality of its data is available. Geometric investigation and calibration of similar range sensors, such as the SwissRanger [19] and PMD [20], has been the topic of several previous works [21–26]. However, these sensors are based on the time-of-flight measurement principle, and are fundamentally different from the Kinect which is a triangulation sensor.

In this paper our primary focus is on the depth data. The objective of the paper is to provide an insight into the geometric quality of the Kinect depth data through calibration and an analysis of the accuracy and density of the points. We present a mathematical model for obtaining 3D object coordinates from the raw image measurements, and discuss the calibration parameters involved in the model. Further, a theoretical random error model is derived and verified by an experiment.

The paper proceeds with a description of the depth measurement principle, the mathematical model and the calibration parameters in Section 2. In Section 3, the error sources are discussed, and a theoretical error model is presented. In Section 4, the models are verified through a number of experiments and the results are discussed. The paper concludes with some remarks in Section 5.

## 2. Depth Measurement by Triangulation

**Figure 1.**
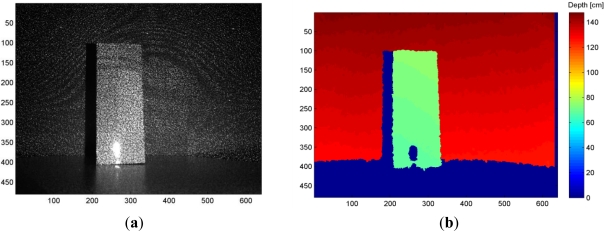
(**a**) Infrared image of the pattern of speckles projected on a sample scene. (**b**) The resulting depth image.

The Kinect sensor consists of an infrared laser emitter, an infrared camera and an RGB camera. The inventors describe the measurement of depth as a triangulation process [27]. The laser source emits a single beam which is split into multiple beams by a diffraction grating to create a constant pattern of speckles projected onto the scene. This pattern is captured by the infrared camera and is correlated against a reference pattern. The reference pattern is obtained by capturing a plane at a known distance from the sensor, and is stored in the memory of the sensor. When a speckle is projected on an object whose distance to the sensor is smaller or larger than that of the reference plane the position of the speckle in the infrared image will be shifted in the direction of the baseline between the laser projector and the perspective center of the infrared camera. These shifts are measured for all speckles by a simple image correlation procedure, which yields a disparity image. For each pixel the distance to the sensor can then be retrieved from the corresponding disparity, as described in the next section. Figure 1 illustrates the depth measurement from the speckle pattern.

### 2.1. Mathematical Model

**Figure 2.**
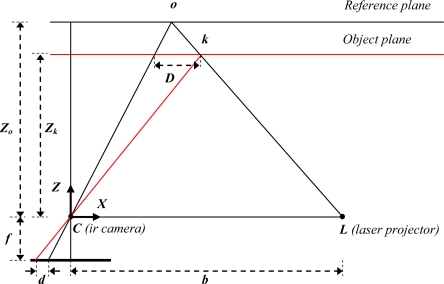
Relation between relative depth and measured disparity.

Figure 2 illustrates the relation between the distance of an object point *k* to the sensor relative to a reference plane and the measured disparity *d*. To express the 3D coordinates of the object points we consider a depth coordinate system with its origin at the perspective center of the infrared camera. The *Z* axis is orthogonal to the image plane towards the object, the *X* axis perpendicular to the *Z* axis in the direction of the baseline *b* between the infrared camera center and the laser projector, and the *Y* axis orthogonal to *X* and *Z* making a right handed coordinate system.

Assume that an object is on the reference plane at a distance *Z_o_* to the sensor, and a speckle on the object is captured on the image plane of the infrared camera. If the object is shifted closer to (or further away from) the sensor the location of the speckle on the image plane will be displaced in the *X* direction. This is measured in image space as disparity *d* corresponding to a point *k* in the object space. From the similarity of triangles we have:

(1) $$\frac{D}{b}=\frac{Z_{o}-Z_{k}}{Z_{o}}$$

and:

(2) $$\frac{d}{f}=\frac{D}{Z_{k}}$$

where *Z_k_* denotes the distance (depth) of the point *k* in object space, *b* is the base length, *f* is the focal length of the infrared camera, *D* is the displacement of the point *k* in object space, and *d* is the observed disparity in image space. Substituting *D* from Equation (2) into Equation (1) and expressing *Z_k_* in terms of the other variables yields:

(3) $$Z_{k}\frac{Z_{o}}{1+\frac{Z_{o}}{\mathrm{fb}}\;d}$$

Equation (3) is the basic mathematical model for the derivation of depth from the observed disparity provided that the constant parameters *Z_o_*, *f*, and *b* can be determined by calibration. The *Z* coordinate of a point together with *f* defines the imaging scale for that point. The planimetric object coordinates of each point can then be calculated from its image coordinates and the scale:

(4) $$\begin{array}{l} X_{k}=-\frac{Z_{k}}{f}\;(x_{k}-x_{o}+\delta x)\\ Y_{k}=-\frac{Z_{k}}{f}\;(y_{k}-y_{o}+\delta y) \end{array}$$

where *x_k_* and *y_k_* are the image coordinates of the point, *x_o_* and *y_o_* are the coordinates of the principal point, and *δx* and *δy* are corrections for lens distortion, for which several models with different coefficients exist; see for instance [28]. Note that here we assume that the image coordinate system is parallel with the base line and thus with the depth coordinate system.

### 2.2. Calibration

As mentioned above, the calibration parameters involved in the mathematical model for the calculation of 3D coordinates from the raw image measurements include:

- focal length (*f*);
- principal point offsets (*x_o_*, *y_o_*);
- lens distortion coefficients (in *δx*, *δy*);
- base length (*b*);
- distance of the reference pattern (*Z_o_*).

In addition, we may consider a misalignment angle between the *x*-axis of the image coordinate system and the base line. However, this does not affect the calculation of the object coordinates if we define the depth coordinate system to be parallel with the image coordinate system instead of the base line. We may, therefore, ignore this misalignment angle.

From the calibration parameters listed above the first three can be determined by a standard calibration of the infrared camera. In practice, however, the calibration parameters of the infrared camera do not directly correspond to the disparity images, because the size of the disparity images computed by the internal processor of Kinect is 640 × 480 pixels, which is smaller than the actual size of the infrared sensor (1,280 × 1,024 pixels) [29]. Due to the bandwidth limitation of the USB connection, the images of the infrared sensor are also streamed in a reduced size of 640 × 480 pixels corresponding to the disparity images (that is the images are resized and cropped).

Therefore, a convenient approach to the calibration is to estimate the calibration parameters from the reduced infrared images instead of the actual sensor, provided that a pixel-to-pixel correspondence exists between the reduced infrared images and the disparity images. By examining the images we observed a shift of 4 pixels in the *x* direction between the disparity and infrared images (supposedly implying the application of a 9-pixel wide correlation window for calculating disparities [30]). Once this shift is corrected for, the calibration parameters estimated from the reduced infrared images can be applied to the measurements in the disparity images.

The determination of the base length and the reference distance is more complicated for the following reason. In practice, the raw disparity measurements are normalized and quantized between 0 and 2,047, and streamed as 11 bit integers. Therefore, in Equation (3)*d* should be replaced with *md’* + *n* with *d’* the normalized disparity and *m*, *n* the parameters of a (supposedly) linear normalization (in fact denormalization). Including these in Equation (3) and inverting it yields:

(5) $$Z_{k}^{-1}=(\frac{m}{\mathrm{fb}})\;d^{\prime }+(Z_{o}^{-1}+\frac{n}{\mathrm{fb}})$$

Equation (5) expresses a linear relation between the inverse depth of a point and its corresponding normalized disparity. By observing the normalized disparity for a number of object points (or planes) at known distances to the sensor the coefficients of this linear relation can be estimated in a least-squares fashion. However, the inclusion of the normalization parameters does not allow determining b and *Z_o_* separately.

The calibration parameters mentioned above completely define the relation between the image measurements (*x*, *y*, *d′*) and object coordinates (*X*, *Y*, *Z*) of each point. Once estimated, they enable the generation of a point cloud from each disparity image. Note that these parameters do not describe the internal geometry of the infrared camera as they are estimated from the resized and cropped images.

### 2.3. Adding Color to the Point Cloud

The integration of the depth and color data requires the orientation of the RGB camera relative to the depth coordinate system. Since we defined the depth coordinate system at the perspective center of the infrared camera we can estimate the orientation parameters by a stereo calibration of the two cameras. The parameters to be estimated include three rotations between the camera coordinate system of the RGB camera and that of the infrared camera, and the 3D position of the perspective center of the RGB camera in the coordinate system of the infrared camera. In addition, the interior orientation parameters of the RGB camera, *i.e.*, the focal length, principal point offsets and the lens distortion parameters must be estimated.

In practice, the images of the RGB camera are also streamed in a reduced size; therefore, it is more convenient to perform a stereo calibration of the reduced images instead of the physical cameras. The resulting parameters describe the relation between the 3D coordinates of each point and its corresponding pixel-coordinates in the reduced RGB image. Once these parameters are estimated, we can add color to the point cloud by projecting each 3D point to the RGB image and interpolating the color.

## 3. Depth Accuracy and Resolution

Accuracy and point density are two important measures for evaluating the quality of a point cloud. In the following sections factors influencing the accuracy and density of Kinect data are discussed, and a theoretical random error model is presented.

### 3.1. Error Sources

Error and imperfection in the Kinect data may originate from three main sources:

- The sensor;
- Measurement setup;
- Properties of the object surface.

The sensor errors, for a properly functioning device, mainly refer to inadequate calibration and inaccurate measurement of disparities. Inadequate calibration and/or error in the estimation of the calibration parameters lead to systematic error in the object coordinates of individual points. Such systematic errors can be eliminated by a proper calibration as described in the previous section. Inaccurate measurement of disparities within the correlation algorithm and particularly the quantization of the disparities also influence the accuracy of individual points.

Errors caused by the measurement setup are mainly related to the lighting condition and the imaging geometry. The lighting condition influences the correlation and measurement of disparities. In strong light the laser speckles appear in low contrast in the infrared image, which can lead to outliers or gap in the resulting point cloud. The imaging geometry includes the distance to the object and the orientation of the object surface relative to the sensor. The operating range of the sensor is between 0.5 m to 5.0 m according to the specifications, and, as we will see in the following section, the random error of depth measurement increases with increasing distance to the sensor. Also, depending on the imaging geometry, parts of the scene may be occluded or shadowed. In Figure 1, the right side of the box is occluded as it cannot be seen by the infrared camera though it may have been illuminated by the laser pattern. The left side of the box is shadowed because it is not illuminated by the laser but is captured in the infrared image. Both the occluded areas and shadows appear as gaps in the point cloud.

The properties of the object surface also impact the measurement of points. As it can be seen in Figure 1, smooth and shiny surfaces that appear overexposed in the infrared image (the lower part of the box) impede the measurement of disparities, and result in a gap in the point cloud.

### 3.2. Theoretical Random Error Model

Assuming that in Equation (5) the calibration parameters are determined accurately and that *d′* is a random variable with a normal distribution we can propagate the variance of the disparity measurement to obtain the variance of the depth measurement as follows:

(6) $$\sigma_{Z}^{2}={\left(\frac{\partial Z}{\partial d}\right)}^{2}\;\sigma_{d^{\prime }}^{2}$$

After simplification this yields the following expression for the standard deviation of depth:

(7) $$\sigma_{Z}=(\frac{m}{\mathrm{fb}})\;Z^{2}\;\sigma_{d^{\prime }}$$

with *σ_d′_* and *σ_Z_* respectively the standard deviation of the measured normalized disparity and the standard deviation of the calculated depth. Equation 7 basically expresses that the random error of depth measurement is proportional to the square distance from the sensor to the object. Since depth is involved in the calculation of the planimetric coordinates, see Equation (4), we expect the error in *X* and *Y* to be also a second order function of depth. By propagating the errors in Equation (4), and assuming that the random error of image coordinates *x*, *y* can be ignored, we obtain the random error of *X* and *Y*:

(8) $$\begin{array}{l} \sigma_{X}=(\frac{\mathrm{mx}}{f^{2}b})Z^{2}\;\sigma_{d^{\prime }}\\ \sigma_{Y}=(\frac{\mathrm{my}}{f^{2}b})Z^{2}\;\sigma_{d^{\prime }} \end{array}$$

### 3.3. Depth Resolution and Point Density

The resolution of the infrared camera, or more precisely the pixel size of the disparity image, determines the point spacing of the depth data on the *XY* plane (perpendicular to camera axis). Since each depth image contains a constant 640 × 480 pixels, the point density will decrease with increasing distance of the object surface from the sensor. Considering the point density as the number of points per unit area, while the number of points remains constant the area is proportional to the square distance from the sensor. Thus, the point density on the *XY* plane is inversely proportional to squared distance from the sensor.

The depth resolution refers to the minimum depth difference that can be measured, and is determined by the number of bits per pixel used to store the disparity measurements. The Kinect disparity measurements are stored as 11-bit integers, where one bit is reserved to mark the pixels for which no disparity is measured, so-called no-data. Thus, a disparity image contains 1,024 levels of disparity. Since depth is inversely proportional to disparity, the resolution of depth is also inversely related to the levels of disparity. Let *Z*(*d′*) denote depth as a function of normalized disparity *d′*, then depth resolution is simply the depth difference corresponding to two successive levels of disparity; *i.e.*, Δ*_Z_*(*d′*) = *Z*(*d′*) – *Z*(*d′* – 1), and as we learned the differential yields:

(9) $$\Delta_{Z}=(\frac{m}{\mathrm{fb}})Z^{2}$$

Thus, the depth resolution is also a quadratic function of depth, and decreases with increasing distance from the sensor.

## 4. Experiments and Results

Experiments were carried out to first determine the calibration parameters of the sensor and then investigate the systematic and random errors in the depth data. The following sections describe the tests and discuss the results.

### 4.1. Calibration Results

**Figure 3.**
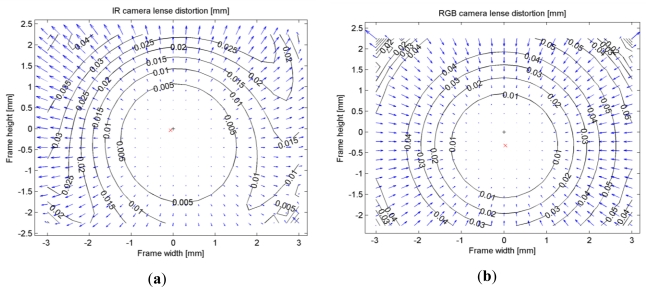
Lens distortions of (**a**) IR camera and (**b**) RGB camera. The principal points are marked by x and the image centers by +.

A standard camera calibration was performed using the reduced images of both the infrared camera and the RGB camera to estimate the calibration parameters in the Photomodeler^®^ software. A total of eight images of a target pattern were taken by both cameras from different angles. To avoid the disturbance of the laser speckles in the infrared images the aperture of the laser emitter was covered by a piece of opaque tape. To model the lens distortion we used the well-known model of Brown [31] with three radial distortion parameters (*K1*, *K2*, *K3*) and two decentering parameters (*P1*, *P2*). The calibration was first performed with all lens distortion parameters as unknowns. Then, those parameters whose standard deviation was large compared to the estimated parameter value were removed from the estimation model, and the remaining parameters were estimated again. As a result, parameter *K3* was excluded from the parameter sets of both cameras, and *P2* was excluded from the parameter set of the RGB camera. Table 1 summarizes the results of the calibration procedure. The overall calibration accuracy as the RMS of point marking residuals in image space was 0.14 pixels for the IR images and 0.09 pixels for the RGB images. In the parameters of the RGB images notice the very large principal point offset (*y_o_*) of 0.327 mm corresponding to 35 pixels; see also Figure 3(b). This value is close to 32 pixels, which is the offset we would expect if a reduced image was obtained by resizing a full resolution image to one-half and cropping it at the top (1024/2 − 480 = 32). The infrared images however do not have large principal point offsets, meaning that they were cropped at the center. The reason for this apparently inconsistent cropping and the large *y_o_* in the RGB images is not known to the authors.

**Figure 4.**
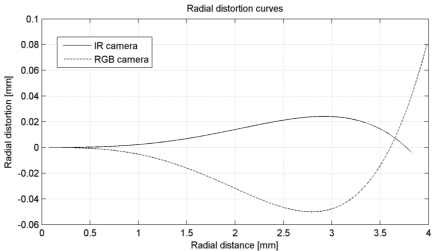
Radial distortion curves for the IR and RGB images.

Figure 3 shows the combined effect of radial and decentering distortions for both the IR and the RGB images. Notice the larger effect of decentering distortions in the IR image as compared to the RGB image. The magnitude of radial distortions however is larger in the RGB image, particularly in the upper left corner where radial distortions reach 9 pixels (0.08 mm). This can be verified by examining the radial distortion curves in Figure 4, which show that radial distortions of the RGB camera are generally larger than those of the IR camera. In practice, radial distortions in the RGB images lead to misalignments between the color and depth data in the point cloud. A distortion of 0.08 mm in the image space corresponds to a misalignment of 8 cm at the maximum range of the sensor (5 m).

For the stereo calibration images of a checkerboard pattern were taken simultaneously by the two cameras, and the relative orientation parameters were estimated in a bundle adjustment. Table 2 lists the resulting parameters. As it can be seen the rotations are quite small, and the relative position parameters indicate that the center of the RGB camera is approximately on the base line between the IR camera and the laser emitter.

**Figure 5.**
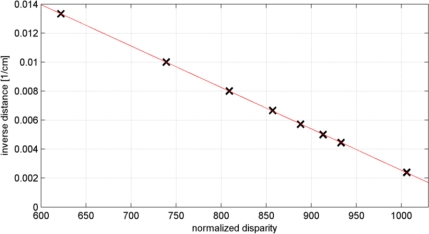
Linear relation of normalized disparity with inverse depth.

To determine the parameters involved in the disparity-depth relation (Equation 5) depth values were measured at eight different distances to the sensor using a measuring tape. The inverse of the measured distances were then plotted against the corresponding normalized disparities observed by the sensor, see Figure 5. As it can be seen the relation is linear as we expected from the mathematical model in Equation (5). The parameters of the disparity-depth relation were obtained by a simple least-squares line regression. These were found to be −2.85e–5 as the slope and 0.03 as the intercept of the line. Using these parameters we can now calculate depth values from the observed normalized disparities.

### 4.2. Comparison of Kinect Point Cloud with the Point Cloud of a High-End Laser Scanner

To investigate the systematic errors in Kinect data a comparison was made with a point cloud obtained by a high-end laser scanner. The Kinect point cloud was obtained from the disparity image using Equations (4) and (5) and the calibration parameters from the previous step. The laser scanner point cloud was obtained of the same scene by a calibrated FARO LS 880 laser scanner. The nominal range accuracy of the laser scanner is 0.7 mm for a highly reflective target at a distance of 10 m to the scanner [32,33]. The average point spacing of the laser scanner point cloud on a surface perpendicular to the range direction (and also the optical axis of the infrared camera of Kinect) was 5 mm. It was therefore assumed that the laser scanner point cloud is sufficiently accurate and dense to serve as reference for the accuracy evaluation of the Kinect point cloud. In the absence of any systematic errors the mean of discrepancies between the two point clouds is expected to be close to zero.

To enable this analysis, first, an accurate registration of the two point clouds is necessary. The registration accuracy is important because any registration error may be misinterpreted as error in the Kinect point cloud. To achieve the best accuracy two registration methods were tested. The first method consisted of a manual rough alignment followed by a fine registration using the iterative closest point (ICP) algorithm [34]. To make ICP more efficient a variant suggested by Pulli [35] was followed in which 200 randomly selected correspondences (closest points) with a rejection rate of 40% were used. In the second method the two roughly-aligned point clouds were segmented into planar surfaces and 20 corresponding segments were manually selected. Then, a robust plane fitting using RANSAC [36,37] was applied to obtain plane parameters and the inlying points. The registration was then performed by minimizing the distances from the points in one point cloud to their corresponding planes in the other point cloud [38].

In both registrations the estimated transformation parameters consisted of a 3D rotation and a 3D translation. To reveal a possible scale difference between the point clouds a third registration was performed using the plane-based method augmented with a scale parameter.

Table 3 summarizes the registration residuals pertaining to the three methods. It is clear that the methods perform similarly, all yielding very comparable residuals. Furthermore, the scale parameter obtained from the third registration was found to be 1.01. The largest effect of such a scale on the furthest point of the point cloud is 5 cm, which is not larger than the random error and depth resolution of the data as will be shown later. Thus, we can conclude that there is no scale difference between the Kinect point cloud and the laser scanner point cloud.

**Figure 6.**
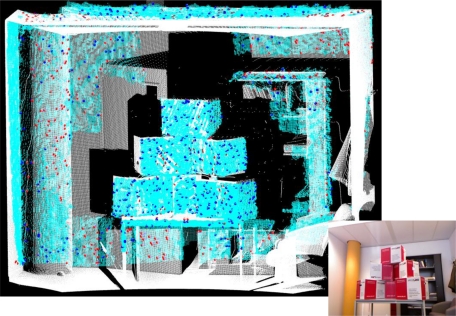
Comparison of Kinect point cloud (**cyan**) with the point cloud obtained by FARO LS880 laser scanner (**white**). The larger points are samples randomly selected from the Kinect data (**blue**) and their closest point in the laser scanner data (**red**). The thumbnail on the lower right is a color image of the setup.

For the comparison between the two point clouds the result of the ICP registration method was used. A total of 1,000 points were randomly selected from the Kinect point cloud and for each point the nearest neighbor was found in the laser scanner point cloud. These closest point pairs were the basis for evaluating the accuracy of the Kinect point cloud. However, it was considered that the point pairs might contain incorrect correspondences, because the two sensors had slightly different viewing angles, and therefore, areas that could not be seen by one sensor might be captured by the other and vice versa. Figure 6 shows the two point clouds and the closest point pairs.

**Figure 7.**
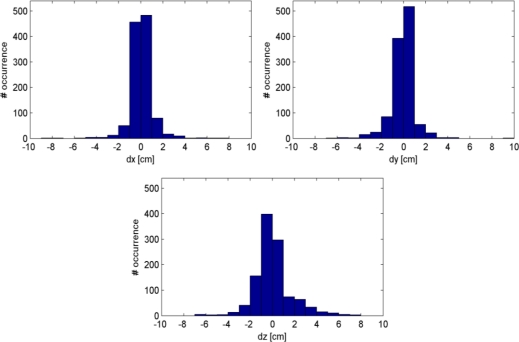
Histograms of discrepancies between the closest point pairs in X, Y and Z direction.

Figure 7 shows the histograms of discrepancies between the point pairs in *X*, *Y* and *Z*. Table 4 lists the statistics related to these discrepancies. The mean and median discrepancies are close to zero, which is an indication that there are no systematic shifts between the two point clouds. For comparison, the last three rows of Table 4 show the discrepancies between the laser scanner point cloud and an uncalibrated Kinect point cloud, measured on the same number of sampled point pairs. The discrepancies are clearly larger when the uncalibrated point cloud is used, indicating the effect of calibration.

**Figure 8.**
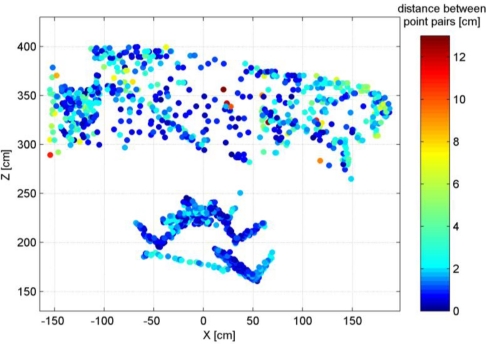
Distribution of point pair distances in the X-Z plane.

Figure 8 shows the distribution of the point pair distances in the *X*-*Z* plane. In general, points that are located further away from the sensor, particularly those at the sides of the point cloud, show larger discrepancies. This is what we expected based on the theoretical random error model. Overall, the comparison of the two point clouds shows that about 84% of the point pairs are less than 3 cm apart.

### 4.3. Plane Fitting Test

To verify the relation between the random error and the distance to the sensor a plane fitting test was carried out. The planar surface of a door was measured at various distances from 0.5 m to 5.0 m (the operation range of the sensor) with 0.5 m intervals.

**Figure 9.**
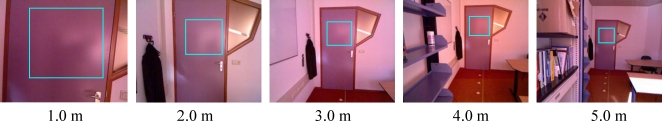
The planar surface of a door measured at different distances to the sensor. The boxes show the plane fitting area.

In each resulting point cloud a same part of the door was selected and a plane was fitted to the selected points. The RANSAC plane fitting method was used to avoid the influence of outliers. Figure 9 shows the measurement setup.

**Figure 10.**
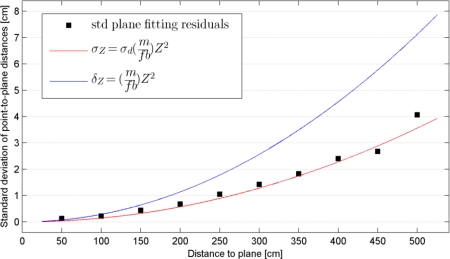
Standard deviation of plane fitting residuals at different distances of the plane to the sensor. The curves show the theoretical random error (**red**) and depth resolution (**blue**).

Since in all measurements the selected planar surface was approximately perpendicular to the optical axis of the sensor the residuals of the plane fitting procedure can be seen as a representation of the depth random error. To evaluate this random error at different distances an equal number of samples (4,500 samples) were randomly selected from each plane, and the standard deviation of the residuals was calculated over the selected samples. Figure 10 shows the calculated standard deviations plotted against the distance from the plane to the sensor (the black squares). It can be seen that the errors increase quadratically from a few millimeters at 0.5 m distance to about 4 cm at the maximum range of the sensor. Although the plane fitting residuals can be seen as an indication of random error of depth measurement, they are also influenced by the depth resolution at each plane. Having determined the calibration parameters we can now evaluate Equations (7) and (9) to obtain the theoretical random error and resolution of individual depth measurements at different distances from the sensor. In Figure 10, the red curve shows the theoretical random error obtained from Equation (7) with | *m*/*fb* | = 2.85e–5 from the depth calibration result (Figure 5) and assuming a disparity measurement error (*σ_d′_*) of ½ pixel. The blue curve is a plot of depth resolution obtained by evaluating Equation (9). The disparity error of ½ pixel seems a fair estimate since the theoretical random error curve is consistent with the observed standard deviations, considering that the low depth resolution has a minor effect on the estimate of the standard deviation of plane fitting residuals when a large number of samples are used.

**Figure 11.**
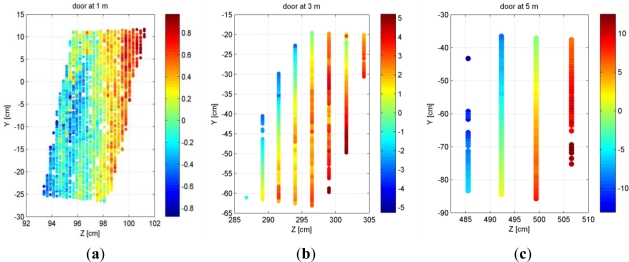
Point cloud of a planar surface at 1 meter (**a**), 3 meter (**b**) and 5 meter (**c**) distance from the sensor projected on the Y-Z plane. Colors represent distance to the best-fit plane in centimeters.

Although depth resolution does not have a large influence on the standard deviation of plane fitting residuals, its effect on the level of individual points should not be understated. This effect is more pronounced at larger distances from the sensor such that at the maximum range of 5 meters the point spacing in the depth direction is 7 centimeters. The combined effect of the random error and low depth resolution at large distances results in a planar surface (perpendicular to the sensor) appearing as several layers of points in the data when seen from side-view. Figure 11 shows the point clouds of the door plane at three distances projected on the Y-Z plane (Z being the depth direction; see Section 2.1 for the definition of the coordinate system). While at 1 m the point spacing in the depth direction is quite small (about 2 mm), at 3 m and 5 m the points are clearly distributed in several layers at intervals corresponding to the depth resolution, which is about 2.5 cm for the plane at 3 m distance and close to 7 cm at 5 m.

## 5. Conclusions

The paper presented a theoretical and experimental analysis of the geometric quality of depth data acquired by the Kinect sensor. The geometric quality measures represent the depth accuracy and resolution for individual points. Indoor mapping applications are often based on the extraction of objects instead of an irregular set of points. In order to describe the quality of extracted objects, some basic error propagation would be needed. While fitting geometric object models to the data can reduce the influence of random errors and low depth resolution, the effect of systematic errors can only be eliminated through a proper calibration procedure.

From the results of calibration and error analysis the following main conclusions can be drawn:

- To eliminate distortions in the point cloud and misalignments between the colour and depth data an accurate stereo calibration of the IR camera and the RGB camera is necessary;
- The random error of depth measurements increases quadratically with increasing distance from the sensor and reaches 4 cm at the maximum range of 5 meters;
- The depth resolution also decreases quadratically with increasing distance from the sensor. The point spacing in the depth direction (along the optical axis of the sensor) is as large as 7 cm at the maximum range of 5 meters.

In general, for mapping applications the data should be acquired within 1–3 m distance to the sensor. At larger distances, the quality of the data is degraded by the noise and low resolution of the depth measurements.

## Figures and Tables

**Table 1.** Calibration parameters estimated for the infrared and RGB images.

**Calibration parameter**		**IR images**	**RGB images**
Focal length	*f*	5.453 ± 0.012 [mm]	4.884 ± 0.006 [mm]
Principal point offset	*x_o_*	−0.063 ± 0.003 [mm]	0.032 ± 0.002 [mm]
	*y_o_*	−0.039 ± 0.008 [mm]	−0.327 ± 0.005 [mm]
Frame dimension	*w*	5.996 ± 0.001 [mm]	6.012 ± 0.002 [mm]
	*h*	4.5 [mm]	4.5 [mm]
Pixel size	*p_x_*	9.3 [μm]	9.3 [μm]
	*p_y_*	9.3 [μm]	9.3 [μm]
Radial lens distortion	*K*1	2.42e–3 ± 1.2e–4	−5.75e–3 ± 6.4e–5
	*K*2	−1.70e–4 ± 1.2e–5	4.42e–4 ± 5.8e–6
	*K*3	0	0
Decentring lens distortion	*P*1	−3.30e–4 ± 3.7e–5	−1.07e–4 ± 2.8e–5
	*P*2	5.25e–4 ± 7.5e–5	0

**Table 2.** Exterior orientation parameters of the RGB camera relative to the IR camera.

**Rotation parameters [degree]**			**Position parameters [mm]**		

***r_x_***	***r_y_***	***r_z_***	***t_x_***	***t_y_***	***t_z_***
0.56	0.07	0.05	−25.60	0.34	2.91

**Table 3.** Registration results of the three methods.

	**Transformation parameters**			**Residuals**				

	*s*	*r_x_, r_y_, r_z_* [deg]	*t_x_, t_y_, t_z_* [cm]	Min [cm]	Mean [cm]	Med [cm]	Std [cm]	Max [cm]
point-point distances (icp)	-	−88.16, 0.03, 0.07	1.20, −0.81, 3.56	0.1	1.2	0.9	0.9	4.4
point-plane distances without scale	-	−91.52, 0.14, −0.23	0.07, −0.32, 0.82	0.0	1.1	0.8	0.9	7.1
point-plane distances with scale	1.01	−90.64, −0.02, 0.04	−0.27, −3.53, −5.60	0.0	1.1	0.9	0.9	7.0

**Table 4.** Statistics of discrepancies between the closest point pairs. The last three rows show, for comparison, the same statistics obtained for an uncalibrated Kinect point cloud.

	**Mean [cm]**	**Median [cm]**	**Standard deviation [cm]**	**Interquartile range [cm]**	**Percentage in [−0.5 cm, 0.5 cm]**	**Percentage in [−1.0 cm, 1.0 cm]**	**Percentage in [−2.0 cm, 2.0 cm]**
dx	0.1	0.0	1.0	0.6	63.4	83.4	95.0
dy	0.0	0.0	1.1	0.6	63.4	80.7	93.2
dz	0.1	−0.1	1.8	1.3	38.9	61.6	82.1
dx *	−0.5	−0.2	1.4	1.0	55.0	74.3	90.9
dy *	−0.6	−0.1	1.5	1.1	56.8	72.7	82.9
dz *	−0.1	−0.4	1.8	1.8	25.1	51.6	81.2
